# The Prophylactic Effect of Acupuncture for Migraine Without Aura: A Randomized, Sham‐Controlled, Clinical Trial

**DOI:** 10.1111/jebm.70059

**Published:** 2025-08-21

**Authors:** Mingsheng Sun, Chaorong Xie, Yanan Wang, Xuguang Yang, Linlin Dong, Taipin Guo, Xiaoqin Chen, Jing Luo, Yutong Zhang, Xixiu Ni, Lu Liu, Jiao Chen, Siyuan Zhou, Ling Zhao

**Affiliations:** ^1^ The Acupuncture and Tuina School Chengdu University of Traditional Chinese Medicine Chengdu China; ^2^ Sichuan Clinical Medical Research Center of Acupuncture and Moxibustion Chengdu China; ^3^ Department of Pain Diseases Hospital of Chongqing of Traditional Chinese Medicine Chongqing China; ^4^ Acupuncture and Tuina Department The Third Affiliated Hospital of Henan University of Traditional Chinese Medicine Zhengzhou China; ^5^ Department of Neurology Hospital of Sichuan Provincial People's Chengdu China; ^6^ The Acupuncture and Tuina School Yunnan University of Traditional Chinese Medicine Kunming China; ^7^ Rehabilitation Department Chengdu Pidu District Hospital of Traditional Chinese Medicine Chengdu China; ^8^ Department of Acupuncture, Tuina and Rehabilitation Southwest Medical University Affiliated Hospital of Traditional Chinese Medicine Luzhou China

**Keywords:** manual penetrating acupuncture, migraine without aura, non‐penetrating acupuncture, randomized controlled trial

## Abstract

**Objective:**

Acupuncture is recognized as an effective migraine treatment, but the comparative long‐term efficacy of different acupuncture methods at identical acupoints remains unclear. This study investigates the prophylactic effects of manual acupuncture (manual penetrating acupuncture, MPA) versus sham acupuncture (non‐penetrating acupuncture, NPA) at the same acupoints.

**Methods:**

In this multicenter, single‐blind randomized controlled trial conducted across four Chinese clinical centers (May 2020 to September 2022), 192 migraineurs without aura (International Classification of Headache Disorders 3rd edition criteria) were randomized 1:1 to 12 sessions of MPA or NPA. Primary outcome was the change from baseline in migraine attack frequency at week 16; secondary outcomes included migraine attack frequency, responder rates, migraine days, and pain intensity (every 4 weeks), etc. Trial registration: *No*. ChiCTR2000032308.

**Results:**

A total of 198 participants were randomly allocated to either MPA or NPA groups, 99 in each group. At 16 weeks, the change in MPA showed a greater reduction in migraine attacks versus NPA (mean difference [MD] = –0.6, 95% confidence interval [CI] –1.5 to 0.05; *p =* 0.069). MPA demonstrated superior responder rates (risk difference = 17.2%, 95% CI 5.2 to 29.1; *p =* 0.007) and pain reduction (MD = –0.6, 95% CI –1.1 to –0.2; *p =* 0.003) after treatment. At follow‐up, MPA improved all migraine symptoms and some quality of life compared with NPA. Adverse events occurred in 5.1% of MPA participants.

**Conclusions:**

Although MPA and NPA showed comparable preventive effects, MPA provided sustained symptom relief and quality‐of‐life improvements. Therefore, suitable acupoint selection establishes therapeutic potential, whereas acupuncture methods critically determine long‐term clinical benefits.

## 1 Introduction

Migraine is a paroxysmal neurological disorder characterized by recurrent and disabling attacks [1, 2]. According to the World Health Organization and the Global Burden of Disease Study, migraine is the third most common disease in the world and the second leading cause of disability [3]. The annual prevalence rate of migraine is approximately 14.4% globally [1, 3] and 9.3% in China. In the United States and Canada, the economic burden of episodic migraines patients exceeds $383 over 3 months [4]. The most commonly prescribed medications for migraine prevention include antiepileptic (e.g., topiramate), beta‐blockers (such as propranolol), and tricyclic antidepressants (including amitriptyline). However, these agents are often associated with adverse effects such as cognitive impairment, fatigue, somnolence, weight gain, and, particularly concerning for the predominantly female patient population, teratogenic risks [5]. Additionally, approximately one to two‐thirds of migraine patients experience medication overuse [6], which further aggravates the frequency of headache attacks [7].

Acupuncture is a well‐established non‐pharmacological treatment for migraine, and a Cochrane systematic review has shown that the use of acupuncture in migraine attacks reduces headache frequency, and that acupuncture may be at least as effective as prophylactic medication [8]. Clinical studies have further shown that manual acupuncture is superior to non‐penetrating at non‐acupoints in preventing migraine attacks [9]. Our previous research has also confirmed the long‐term prophylactic effects of acupuncture compared to non‐acupoint [10]. In 2022, the *BMJ* has published a series of articles analyzing the current state of acupuncture research and suggesting that the insertion of needles and even skin contact produces specific effects, creating the possibility that current trials using sham acupuncture may be generally underestimating the therapeutic effects of thereof [11, 12]. Similarly, the lack of research on different methods at acupoints in migraine studies may contribute to an incomplete understanding of the nonspecific effects of acupuncture. To address this gap, we conducted a randomized controlled trial (RCT) comparing manual acupuncture (manual penetrating acupuncture, MPA) with sham acupuncture (non‐penetrating acupuncture, NPA) at the same acupoints, aiming to comprehensively evaluate the clinical efficacy of acupuncture in migraine prophylaxis.

## 2 Methods

The study protocol was approved by the ethics committee of the Hospital of Chengdu University of Traditional Chinese Medicine (TCM) prior to participants enrollment (*No*. 2020KL‐003). Written informed consent was obtained from all participants before randomization. This trial was registered with Chinese Clinical Trial Registry (*No*. ChiCTR 2000032308). The study adhered to the Declaration of Helsinki and the Consolidated Standards of Reporting Trials (CONSORT) guidelines [13, 14].

### 2.1 Study Design

This was a multicenter, parallel‐group, superiority, single‐blind RCT. Participants were recruited from the outpatient units of the Departments of Acupuncture and Neurology at four clinical centers: the hospital of Chengdu University of TCM, Southwest Medical University Affiliated Hospital of TCM, Chengdu Pidu District Hospital of TCM, and the third affiliated Hospital of the Henan University of TCM, from May 2020 to September 2022.

### 2.2 Eligibility Criteria

The inclusion criteria were as follows: (a) men or women aged 18 to 55 years old with initial onset of migraines prior to the age of 50 years; (b) meeting the migraine without aura diagnostic criteria in the International Classification of Headache Disorders 3rd edition (ICHD‐3) [15] and the guidelines of the International Headache Society for controlled trials of preventive treatment of migraine attacks in adults with episodic migraine [16]; (c) had had 2 to 8 migraine attacks, but less than 15 days of attacks per month, and have not received any acupuncture treatment during the last 3 months and baseline; (d) who had experienced migraine attacks for at least 1 year; (e) with a visual analog scale (VAS) score of 3 to 7 during the baseline period; (f) completed headache diary and provided baseline values in the diary; and (g) provision of written, informed consent.

Patients were excluded if they had tension‐type headache, cluster headache, or other primary headaches; secondary headache disorders; neuralgia of the face or head; relatively severe systemic illness; severe mental illness; pregnancy, lactation, or inadequate contraception; involvement in other clinical trials; and an inability to read and understand the evaluation scales. The procedures were conducted as per a previous study we published [17].

### 2.3 Interventions

All acupuncturists had at least 5 years of training and were licensed with a minimum of 5 years of experience. Patients in both groups received 12 sessions of acupuncture treatment (three treatment sessions per week), each lasting 30 min, for 4 weeks. Migraineurs were not allowed to take any prophylactic medications. In cases of intolerable headaches, the patients were instructed to take ibuprofen (300 mg capsules with sustained release, Zhuhai Embellish Pharmaceutical co., LTD) as a rescue medication, and the usage of ibuprofen was documented in the headache diary [10].

Five acupoints were used per treatment. All patients received acupuncture on three obligatory points, including GV20, GB20, and GB8. The other two points were chosen according to the syndrome differentiation of meridians in the headache region (Figure S1 in Supplement Materials). The use of additional acupoints other than those prescribed was not allowed. We chose the prescriptions as a result of TCM theory, consensus meetings with clinical experts, and the results of our previous research [10].

The Park sham acupuncture device (Dong‐bang Acupuncture, Seoul, Korea) was used for both MPA and NPA. Single‐use filiform acupuncture needles, each with a length of 25–40 mm and a diameter of 0.25 mm, were used in the MPA group. Each point was acupunctured to achieve the *Deqi* sensation (a sensation of soreness, numbness, distention, or radiating) [18], and needling manipulation (rotation or lifting for 10 s) was performed every 15 min to evoke a *Deqi* sensation. In the NPA group, the needle tip is blunt, and when it touches the skin, a pricking sensation is felt by the patient, simulating the puncturing of the skin.

### 2.4 Outcomes

During the course of the study, after team discussion and deliberation, we revised the primary outcome to the change in the frequency of migraine attacks during the 16 weeks after randomization compared with baseline. The modification of our primary outcome to a single 16‐week endpoint was driven by dual considerations: the chronicity and relapse patterns of migraine, and statistical optimization to minimize false‐positive risks from multiple comparisons. This approach enhances the reliability and clinical translatability of our efficacy evidence. Secondary outcomes included the frequency of migraine attacks, proportion of responders (defined as the proportion of patients with at least a 50% reduction in the number of migraine attacks), days with migraine, average headache severity (evaluated by VAS), change in the Headache Impact Test‐6 (HIT‐6) and Migraine‐Specific Quality of Life Questionnaire, intake dose of rescue medication every 4 weeks. Changes in serum calcitonin gene‐related peptide (CGRP) at baseline, week 4, and week 16, and evaluate patient satisfaction with treatment at the end of the study. In addition, to test the blinding for group allocation, patients were also asked at the end of the study to guess which type of acupuncture therapy they had received. Researchers documented acupuncture treatment and reasons for dropouts during the study period. Acupuncture‐associated adverse events (AEs), including bleeding, a subcutaneous hemorrhage, hematoma, fainting, serious pain, and local infection, were recorded at each treatment session.

### 2.5 Sample Size

Based on our previous study [19], we anticipated a mean clinically relevant difference of 1 between the two groups over 16 weeks, a mean frequency of migraine attacks of 2 in the MPA group and 3 in the NPA group, and a standard deviation (SD) of 2. With a two‐sided significance level of 5% and power of 90%, 86 patients were required for each group. In the published protocol, we initially calculated the sample size based on a 15% dropout rate. However, during the pilot phase, we adjusted the dropout rate to 10% due to high participant adherence and optimized follow‐up conditions, recalculating the sample size to 96 patients per group. During the study, participants were highly motivated, and finally 99 patients were included in each group (An application to expand the recruitment sample size was submitted to and approved by the ethics committee), which enhanced the statistical power and reliability of our results.

### 2.6 Randomization and Blinding

Eligible participants were dynamically randomized assigned in a 1:1 ratio to receive MPA or NPA, the random sequence was generated and masked by the online messaging system of Chengdu Clinical Information Management System (CIMS) Medical Technology Co., Ltd. Different sub‐centers were set as stratification factors, with block lengths of 4–6, which was masked to the investigators. The clinical research assistants at each hospital sent randomization information (including center number, patient name pinyin initials, and screening number) to the CIMS center via online system or mobile phone message, requesting randomization sequence and group. The randomization sequence will be concealed in the server of CIMS until this study finishes participant enrollment, observation and data collection. Participants were blinded to the acupoints selection in both groups, but the acupuncturist could not be blinded to treatment assignments given the particularity of the acupuncture procedure. Outcome assessors, data collectors, and statisticians were blinded to the treatment allocation.

### 2.7 Data Collection

Participants were instructed to complete headache diaries every 4 weeks after enrollment. The diary was used to document the time of migraine onset, duration, severity (evaluated by VAS score), and rescue medication use. Serum CGRP was collected at baseline, 4 weeks and 16 weeks in Chengdu. At each follow‐up, two blinded evaluators at each clinical center reminded patients via phone call, text message, or WeChat to return the headache diary to the trial office through email or to outpatient offices at follow‐up visits.

### 2.8 Statistical Analysis

Intent‐to‐treat (ITT) analyses were conducted using complete case data, including participants with at least 1 primary outcome assessment. We used the multiple imputation by chained equations algorithm to impute 5 distinct datasets for both the primary and secondary outcomes. These 5 data sets were subsequently merged into a single data set for further analysis. Continuous data are summarized as means and SDs or mean difference and 95% CI, and binary or ordinal data are described as numbers and percentages or risk difference and 95% CI. In the outcome analysis, for continuous data, we used the *z*‐test to detect differences between the two groups. For dichotomous data, the χ^2^ test or Fisher's exact test was used. Values of *p <* 0.05 were considered to indicate significance. Based on previous studies, we determined the minimal clinical important difference (MCID) measure for some secondary outcome measures (migraine days, 0.5 days [19]; VAS, 1 point [20]; HIT‐6, 2.3 points [21]; MSQ‐role restrictive domain, 3.2 points [22]; MSQ‐role preventive domain, 4.6 points [22]; MSQ‐ emotional functioning domain, 7.5 points [22]). We did a secondary analysis by replicating the analyses in the per‐protocol data set (PPS). The PPS included the participants who adhered to the assigned treatment and the participants who received at least 10 sessions of acupuncture were considered to have credible adherence to the acupuncture protocol. The statistical analysis was performed using IBM SPSS statistical software version 26.0 (IBM Corp., Armonk, NY, USA) and R software (R Foundation), version 4.4.1.

## 3 Results

### 3.1 Participants and Baseline Characteristics

**FIGURE 1:**
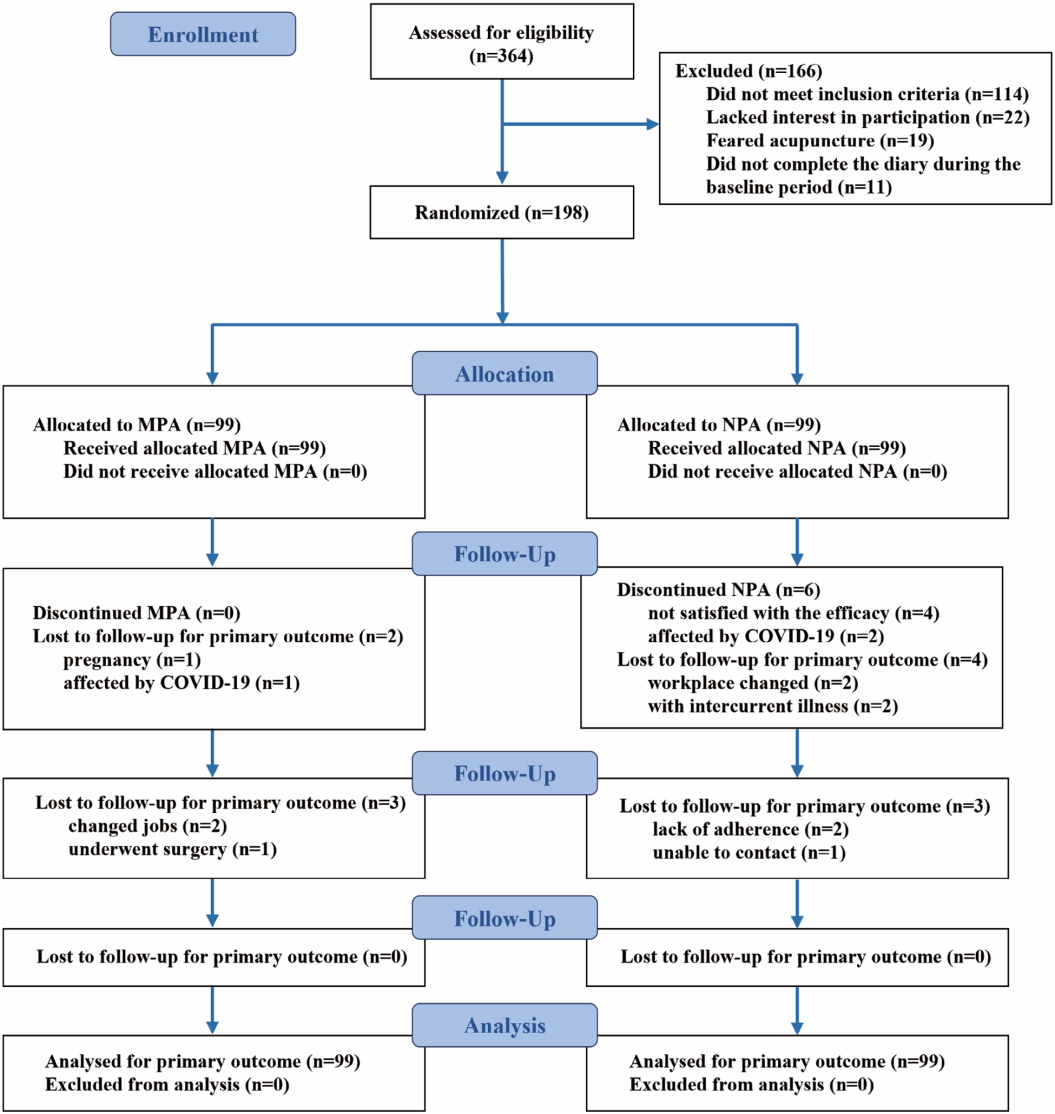
Flowchart of the screening, enrollment, randomization, and follow‐up. MPA, manual acupuncture (manual penetrating acupuncture); NPA, sham acupuncture (non‐penetrating acupuncture); COVID‐19, Corona Virus Disease 2019; ITT, intent‐to‐treat.

A total of 364 patients were screened for eligibility, and 198 (54.4%) participants underwent randomization. A total of 198 patients (mean [SD] age, 35.6 [10.3] years; 150 [75.8%] women) were randomly assigned to either the MPA group (*n* = 99) or the NPA group (*n* = 99) and were included in the ITT population. Figure 1 shows the screening process and the reasons for exclusion. Eighteen patients (9.1%) dropped out (five in the MPA group and 13 in the NPA group). Table 1 shows patient characteristics at baseline and acupuncture expectations before treatment.

**TABLE 1:** Baseline characteristics of 198 patients included in the intent‐to‐treat.

Characteristic	MPA (*n* = 99)		NPA (*n* = 99)
Age, mean (SD), year	35.3 (10.0)	35.9 (10.7)	
Sex, No. (%)			
Male	24 (24.2)	24 (24.2)	
Female	75 (75.8)	75 (75.8)	
BMI (Kg/m^2^), mean (SD)	22.0 (3.2)	22.1 (3.9)	
Duration of illness, mean (SD), month	97.4 (89.3)	112.5 (98.8)	
Degree of education, mean (SD), year	14.5 (3.7)	15.2 (3.5)	
Family history, yes, No. (%)	28 (28.3)	25 (25.3)	
Use of acute pain medication, No. (%)	52 (53.6)	48 (53.9)	
Accompanying symptoms, No. (%)			
Nausea or vomiting	44 (44.4)	42 (42.4)	
Photophobia or phonophobia	62 (62.6)	71 (71.7)	
Others	12 (12.1)	17 (17.2)	
Acupuncture expectation of improvement, No. (%)			
None	0 (0.0)	1 (1.0)	
Slight	4 (4.0)	17 (17.2)	
Some	24 (24.2)	21 (21.2)	
Significant	71 (71.7)	60 (60.6)	

Abbreviations: BMI, body mass index; MPA, manual acupuncture (manual penetrating acupuncture); NPA, sham acupuncture (non‐penetrating acupuncture); SD, standard deviation.

### 3.2 Primary Outcome

**FIGURE 2:**
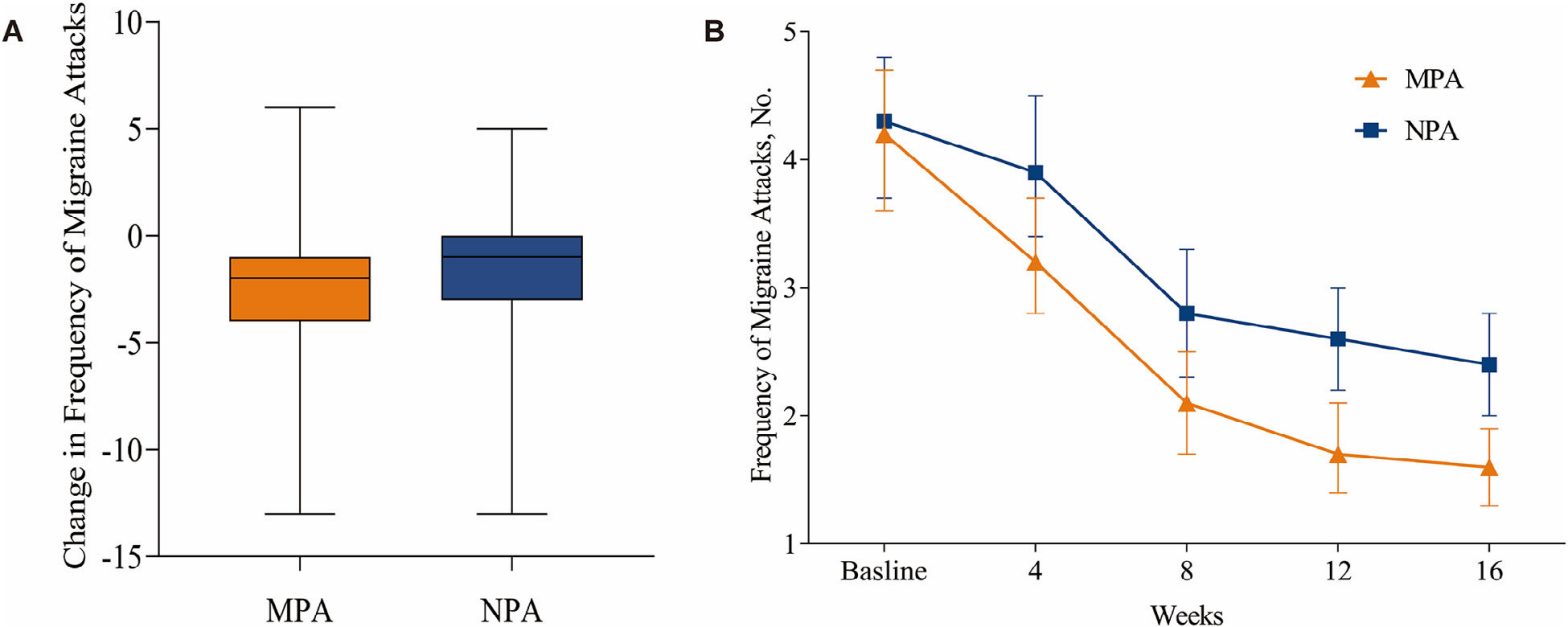
Frequency of migraine attacks. (A) Change in frequency of migraine attacks at 16 weeks; in the box plots, the horizontal lines indicate medians; and the tops and bottoms of the boxes indicate upper and lower quartiles, respectively. (B) Frequency of migraine attacks throughout the study; in the line plot, error bars indicate 95% CIs. MPA, manual acupuncture (manual penetrating acupuncture); NPA, sham acupuncture (non‐penetrating acupuncture); CI, confidence interval.

Table 2 and Figure 2 show the primary analyses. The change in the frequency of migraine attacks for MPA group from baseline (mean [SD], 4.2 [2.5]) to week 16 (mean [SD], 1.6 [1.6]) was –2.6 (SD, 2.8). The change in the frequency of migraine attacks for NPA group from baseline (mean [SD], 4.2 [2.7]) to week 16 (mean [SD], 2.4 [2.0]) was –1.9 (SD, 2.7). The participants who received MPA had a greater reduction in frequency of migraine attacks than those in the NPA group, with a group difference of –0.6 (95% CI, –1.5 to 0.05; *p =* 0.069) attacks, although not met the significant statistical difference. The PPS showed consistent results (Tables S1 and S2 in Supplement Materials).

**TABLE 2:** Headache diary–based outcome measurements during the study.

Variable	MPA (*n* = 99)	NPA (*n* = 99)	Difference, 95% CI	*p* Value
**Primary outcome**				
Change from baseline in migraine attacks frequency at week 16,^a^ mean (SD)				
Week 16	−2.6 (2.8)	−1.9 (2.7)	−0.6 (–1.5 to 0.05)	0.069
**Secondary outcome**				
Frequency of migraine attacks per 4 weeks, mean (SD)				
Baseline	4.2 (2.5)	4.2 (2.7)	−0.1 (–0.8 to 0.6)	0.828
Treatment, week 4	3.7 (2.7)	4.4 (3.2)	−0.6 (–1.5 to 0.08)	0.079
After treatment				
Week 8	2.1 (2.1)	2.8 (2.5)	−0.7 (–1.4 to –0.09)	0.025^*^
Week 12	1.7 (1.8)	2.6 (2.2)	−0.9 (–1.4 to –0.3)	0.002^**^
Week 16	1.6 (1.6)	2.4 (2.0)	−0.8 (–1.3 to –0.3)	0.002^**^
Proportion of responders per 4 weeks, No. (%)				
Treatment, week 4	34 (36.4)	17 (18.2)	17.2 (5.2 to 29.1)	0.007^**^
After treatment				
Week 8	57 (59.6)	35 (44.4)	15.2 (8.7 to 23.8)	0.040^*^
Week 12	64 (67.7)	36 (45.4)	28.3 (14.9 to 41.6)	<0.001^***^
Week 16	67 (68.7)	39 (48.5)	28.3 (15.0 to 41.6)	<0.001^***^
Days with migraine per 4 weeks,^b^ mean (SD)				
Baseline	4.7 (3.2)	4.6 (3.0)	0.1 (–0.7 to 1.0)	0.763
Treatment, week 4	3.7 (2.7)	4.4 (3.2)	−0.7^∆^ (–1.5 to 0.08)	0.079
After treatment				
Week 8	2.4 (2.4)	3.1 (2.9)	−0.7^∆^(–1.5 to –0.0004)	0.049^*^
Week 12	2.0 (2.1)	3.0 (2.3)	−1.0^∆^ (–1.6 to –0.4)	0.001^**^
Week 16	1.8 (2.0)	2.7 (2.2)	−0.9^∆^ (–1.6 to –0.4)	0.001^**^
VAS score per 4 weeks, mean (SD)				
Baseline	5.1 (1.3)	5.3 (1.3)	−0.2 (–0.5 to 0.2)	0.524
Treatment, week 4	3.9 (1.5)	4.5 (1.5)	−0.6 (–1.1 to –0.2)	0.003^**^
After treatment				
Week 8	2.9 (2.0)	4.0 (2.1)	−1.1^∆^ (–1.6 to –0.5)	<0.001^***^
Week 12	2.7 (1.9)	4.0 (2.0)	−1.3^∆^ (–1.8 to –0.7)	<0.001^***^
Week 16	2.7 (2.1)	3.6 (2.1)	−0.9 (–1.5 to –0.3)	0.003^**^
Use of acute pain medication per 4 weeks, No. (%)				
Baseline	45 (46.4)	50 (50.6)	−5.1 (–19.0 to 8.8)	0.478
Treatment, week 4	27 (26.8)	34 (34.8)	−7.1 (–19.9 to 5.8)	0.282
After treatment				
Week 8	20 (20.6)	28 (29.2)	−8.1 (–20.0 to 3.8)	0.186
Week 12	11 (11.3)	24 (27.0)	−13.1 (–23.6 to –2.7)	0.016^*^
Week 16	16 (16.5)	22 (23.6)	−6.1 (–17.0 to 0.05)	0.280
Frequency of acute pain medication use per 4 weeks, mean (SD)				
Baseline	2.4 (2.2)	2.6 (2.8)	−0.3 (–1.2 to 0.7)	0.590
Treatment, week 4	1.2 (1.5)	1.6 (2.5)	−0.5 (–1.2 to 0.3)	0.254
After treatment				
Week 8	0.8 (1.6)	1.1 (1.8)	−0.4 (–1.0 to 0.2)	0.232
Week 12	0.5 (1.5)	1.1 (1.8)	−0.5 (–1.2 to 0.08)	0.088
Week 16	0.6 (1.2)	0.9 (1.6)	−0.3 (–0.9 to 0.2)	0.210

*Note*: Data are given as mean (SD) except where noted.

Abbreviations: CI, confidence interval; MCID, minimal clinical important difference; MPA, manual acupuncture (manual penetrating acupuncture); NPA, sham acupuncture (non‐penetrating acupuncture); SD, standard deviation; VAS, visual analog scale.

^a^ The frequency of migraine attack was defined as the number of episodes of migraine attack separated by pain‐free intervals of at least 48 h, as recorded in the headache diary.

^b^ Number of days with migraine was defined as the duration of migraine attacks.

^∆^ Difference > MCID.

* *p* < 0.05.

** *p* < 0.01.

*** *p* < 0.001.

### 3.3 Secondary Outcomes

At the end of the fourth week, meaningful differences in the proportion of responders and VAS scores were observed between the MPA group and the NPA group, and no differences were found in migraine frequency, migraine days (meet the MCID), changed values of HIT‐6 scores and MSQ scores (role prevention scores in MSQ meet the MCID) (*p >* 0.05) (Tables 2 and 3). In each interview at week 8, 12 and 16, the reduction of migraine frequency, migraine days (meet the MCID), VAS score (meet the MCID at week 8 and 12), and change values in HIT‐6 scores (meet the MCID) were significantly greater in the MPA group than in the NPA group. Additionally, the MPA group showed a higher proportion of responders. We also found significantly higher changed values in role restriction domain (meet the MCID at week 8, 12 and 16) and role prevention scores (meet the MCID at week 8, 12 and 16) in MSQ at week 8 and 12 (Table 2). The number of patients using acute pain medication, such as ibuprofen, significantly differed between the two groups at week 12, and no difference was found in the frequency of use (Table 2).

**TABLE 3:** HIT‐6 score and MSQ score.

Variable	MPA (*n* = 99)	NPA (*n* = 99)	Difference, 95% CI	*p* Value
**Secondary outcomes**				
Change from baseline in HIT‐6 Score, mean (SD)				
Baseline	62.3 (7.2)	62.0 (7.3)	0.30 (–1.8 to 2.3)	0.807
Treatment, week 4	−6.7 (8.4)	−4.6 (7.6)	−2.1 (–4.3 to 0.1)	0.066
After treatment				
Week 8	−11.7 (11.3)	−7.7 (9.9)	−3.9^∆^ (–6.9 to –1.0)	0.009^**^
Week 12	−13.2 (11.3)	−8.2 (9.4)	−4.9^∆^ (–7.8 to –2.0)	<00.001^***^
Week 16	−13.5 (11.3)	−9.9 (9.7)	−3.6^∆^ (–6.5 to –0.6)	0.017^*^
Change from baseline in MSQ score, role restrictive domain, mean (SD)				
Baseline	60.7 (17.2)	60.4 (15.7)	0.3 (–4.2 to 4.9)	0.882
Treatment, week 4	13.4 (19.2)	10.4 (17.8)	3.0 (–2.2 to 8.1)	0.259
After treatment				
Week 8	19.6 (21.1)	13.9 (19.5)	5.7^∆^ (0.9 to 11.3)	0.047^*^
Week 12	20.5 (22.8)	14.1 (17.8)	6.3^∆^ (0.6 to 12.0)	0.030^*^
Week 16	22.2 (23.7)	17.0 (19.3)	5.2^∆^ (–0.8 to 11.2)	0.090
Change from baseline in MSQ score, role preventive domain, mean (SD)				
Baseline	70.4 (19.3)	71.2 (20.2)	−0.8 (–6.4 to 4.6)	0.759
Treatment, week 4	11.7 (20.7)	6.7 (18.6)	5.1^∆^ (–0.4 to 10.5)	0.071
After treatment				
Week 8	15.6 (21.9)	9.6 (19.7)	6.0^∆^ (0.16 to 11.8)	0.044^*^
Week 12	16.7 (22.7)	9.3 (19.6)	7.3^∆^ (1.4 to 13.2)	0.015^*^
Week 16	16.3 (23.3)	10.8 (20.0)	5.5^∆^ (–0.6 to 11.5)	0.076
Change from baseline in MSQ score, emotional functioning domain, mean (SD)				
Baseline	77.8 (16.6)	76.3 (17.7)	1.5 (–3.3 to 6.3)	0.544
Treatment, week 4	8.3 (20.3)	4.7 (16.6)	3.6 (–1.6 to 8.7)	0.177
After treatment				
Week 8	9.7 (19.6)	5.8 (18.3)	3.9 (–1.4 to 9.2)	0.148
Week 12	10.8 (22.5)	6.7 (19.0)	4.2 (–1.6 to 10.0)	0.159
Week 16	10.4 (20.5)	9.2 (19.5)	1.2 (–4.4 to 6.8)	0.670

*Note*: Data are given as mean (SD).

Abbreviations: CI, confidence interval; HIT‐6, Headache Impact Test‐6; MCID, minimal clinical important difference; MPA, manual acupuncture (manual penetrating acupuncture); MSQ, Migraine‐Specific Quality of Life Questionnaire; NPA, sham acupuncture (non‐penetrating acupuncture); SD, standard deviation.

^∆^ Difference>MCID.

* *p <* 0.05.

** *p <* 0.01.

*** *p <* 0.001.

There was no difference in the characteristics of patients measured CGRP at baseline (Table S3 in Supplement Materials), and no significant differences in changes between the two groups at week 4 and week 16 (Table S4 in Supplement Materials). At the end of the trial, patients in the MPA group were significantly more satisfied with treatment than in the NPA group (Table S5 in Supplement Materials). The credibility assessment of blinding showed a significant difference in the correct guess rate between the MPA group and NPA group (Table S6 in Supplement Materials). However, the subgroup analyses of participants with credible blinding showed results were similar to the ITT analysis (Tables S7 and S8 in Supplement Materials).

### 3.4 Adverse Events

Five participants in the MPA group reported AEs during the 4‐week treatment period, and one patient withdrew from the trial due to dizziness after needling. Two patients had the sensation of needle fainting during the first acupuncture treatment, one patient had a subcutaneous hemorrhage in the needle insertion area, and one patient had local pain in the needle insertion area after acupuncture. All AEs were classified as moderate, and none required medical interventions. The remaining four patients fully recovered from the AEs and did not withdraw from the trial.

## 4 Discussion

At week 16, we found no statistically significant difference between MPA and NPA in reducing the change in migraine attack frequency (primary outcome). However, MPA demonstrated superior efficacy over NPA for several key secondary outcomes. For example, greater reductions in migraine attack frequency, higher response rates, fewer migraine days, and reduced pain intensity. These therapeutic benefits emerged at the first post‐treatment follow‐up (week 8) and persisted through the final assessment (week 16). Notably, both migraine days (after treatment and throughout follow‐up) and pain intensity (week 8 and 12) demonstrated clinically meaningful improvements (meet the MCID). Furthermore, our findings indicate that MPA not only alleviates migraine symptoms but also significantly enhances patients’ quality of life, as measured by both the HIT‐6 and the MSQ. The preventive effect of MPA lasted for 16 weeks, with between‐group differences in the changes of HIT‐6 and MSQ score (role restriction domain and role prevention domain) during follow‐up all meet the MCID. To ensure result validity, we maintained detailed documentation of concomitant medications and treatments throughout the study period, thereby controlling for potential confounding effects from other therapies during follow‐up. These findings suggest that while both interventions may share common non‐specific effects, MPA appears to confer additional specific effects, highlighting the importance of penetration method in maintaining long‐term efficacy for migraine prophylaxis.

In this study, NPA at the same acupoints demonstrated short‐term efficacy in reducing migraine frequency and migraine days while improving quality of life during the treatment phase. In contrast, only a small percentage of patients exhibited reductions in migraine days and anxiety scores with non‐acupoint treatment [10, 23, 24]. This differential therapeutic response highlights the essential role of acupoint specificity in achieving clinical efficacy. As non‐pharmacological interventions, non‐penetrating acupoint‐based therapies such as acupoint scraping [25], acupoint cupping [26, 27], and acupressure [28] are widely used in clinical practice and have demonstrated efficacy in RCTs. Therefore, the comparable efficacy between MPA and NPA for primary outcomes suggests two important considerations: first, non‐specific effects contribute substantially to clinical improvement; and second, regardless of the needling technique, acupoint stimulation itself may have inherent specific effects.

Furthermore, MPA demonstrated sustained efficacy across the majority of evaluated metrics throughout weeks 5 to 16. These findings align with prior studies [9, 10, 23], where acupuncture or electroacupuncture was significantly more effective than sham acupuncture (non‐acupoint non‐penetrating method or non‐acupoint minimal stimulation method) during or after follow‐up. In the MPA group, we elicited *Deqi* sensation at each acupoint and maintained it through manual manipulation, ensuring continuous stimulation method. Previous research supports this approach: electroacupuncture was most effective in reducing neck pain, superior to minimal stimulation methods (both acupoint and non‐acupoint), whereas deep puncture at non‐acupoint locations (adjacent to affected muscles) produced analgesic effects comparable to true acupuncture [29]. Similarly, another study found that while both acupuncture and non‐acupoint minimal stimulation reduced anxiety in Parkinson's patients post‐treatment [30], only the acupuncture group maintained significant improvements at follow‐up. These studies indicate that penetrating stimulation method at acupoints and stimulation intensity are key for sustained therapeutic efficacy.

Neuroimaging studies have consistently demonstrated altered functional connectivity (FC) in pain‐processing brain networks among migraine patients [31, 32, 33, 34]. These findings suggest that acupuncture's therapeutic effects in migraine may involve modulation of central pain pathways. Previous exploratory studies of different acupuncture methods at the same acupoint indicate that clinical benefits may derive not only from local tissue stimulation but also from non‐specific mechanisms, including psychological and placebo effects [35]. Experimental evidence shows a direct correlation between distinct acupuncture sensations and subsequent analgesic responses [36]. Functional magnetic resonance imaging reveal that both acupuncture and non‐penetrating method triggered sensations associated with sensorimotor system activation and default‐mode network inactivation [37]. The sensations triggered by acupuncture were qualitatively more complex and long‐lasting, whereas non‐penetrating method was associated with greater activation of the sensorimotor system and stronger inactivation in the default‐mode network. In addition, acupuncture sensation focuses attention and enhances body awareness, contributing to enhanced top‐down modulation of injury sensory afferents and central pain networks. This may be the key that can be used to explain the non‐specific effects of acupuncture.

At the end of the trial, the credibility assessment of blinding revealed partial unblinding in NPA group, consistent with previous reports [38, 39]. We speculate this may relate to participants’ prior acupuncture experience, as untreated patients might better maintain blinding. However, the overall trial results appear unlikely to be substantially biased by this limitation. In addition, due to logistical challenges (e.g., sample degradation during transportation and variability in testing procedures across centers), CGRP samples were only collected and tested in the Chengdu sub‐center, resulting in a smaller sample size. And because CGRP was not mandatory for all participants, some patients in the control group declined post‐treatment sample collection, leading to an imbalance in sample numbers between groups.

Our study has several strengths. For the first time, this represents the first large‐scale comparison of different acupuncture methods applied to same acupoints in migraine without aura patients, providing robust evidence for assessing the specific effect of acupuncture. And by designing long‐term follow‐up, allowing us to differentiate between specific and non‐specific therapeutic effects, offering a basis for the specific therapeutic role of acupuncture. This study has several limitations. First, we did not ask patients about their prior acupuncture experience, which may have affected the blinding effect, particularly for individuals who were not naive to treatment. Second, many studies assessing non‐specific effects suggested that placebo improves symptoms but not objective indicators. Due to the specific nature of pain studies, we also used primarily patient‐reported outcomes. But future studies may need to use more objective indicators to better assess the specific effects of MPA. Third, the study population consisted primarily of patients with mild‐to‐moderate pain (mean baseline VAS: 5.25), which may underestimate the efficacy of MPA, and future studies may consider including patients with moderate to severe pain.

In conclusion, while MPA and the NPA showed comparable reductions in migraine attack frequency at the 16‐week endpoint, MPA showed superior long‐term therapeutic benefits. It provided pain relief during treatment and sustained improvements in migraine‐related disability and quality of life throughout the follow‐up period. These results highlight that acupoint specific effect establishes the foundation for therapeutic efficacy, whereas choice of acupuncture methods is critical for maintaining long‐term clinical benefits.

## Conflicts of Interest

The authors declare no conflicts of interest.

## Supporting information




**Supporting File 1**: jebm70059‐sup‐0001‐SuppMat.docx

## Data Availability

The data underlying this article will be shared on reasonable request to the corresponding author.
