# Supplementary material for: The Prophylactic Effect of Acupuncture for Migraine Without Aura: A Randomized, Sham‐Controlled, Clinical Trial
Source: J Evid Based Med. 2025 Aug 21;18(3):e70059. doi: 10.1111/jebm.70059 (PMC12506941; doi:10.1111/jebm.70059)
Supplement: Supplementary file 1 — Supporting File 1: jebm70059‐sup‐0001‐SuppMat.docx [file JEBM-18-0-s001.docx]

**Supplementary Materials**

[Figure S1. Location of acupoints based on syndrome differentiation of meridians 1](#_Toc200106152)

[Table S1. Outcome measurements of the per-protocol set population: headache diary-based outcome measurements during the entire study 2](#_Toc200106153)

[Table S2. Outcome measurements of the per-protocol set population: headache diary-HIT-6 score and MSQ score 4](#_Toc200106154)

[Table S3. Baseline characteristics of patients with calcitonin gene-related peptide were measured 5](#_Toc200106155)

[Table S4. Change from baseline in calcitonin gene-related peptide 5](#_Toc200106156)

[Table S5. Assessment of satisfaction in the intent-to-treat population 5](#_Toc200106157)

[Table S6. Assessment of blinding of the intent-to-treat population 6](#_Toc200106157)

[Table S7. Outcome measurements - participants with credible blinding: headache diary-based outcome measurements during the entire study 7](#_Toc200106158)

[Table S8. Outcome measurements - participants with credible blinding: headache diary -HIT-6 score and MSQ score 9](#_Toc200106159)

# Figure S1. Location of acupoints based on syndrome differentiation of meridians


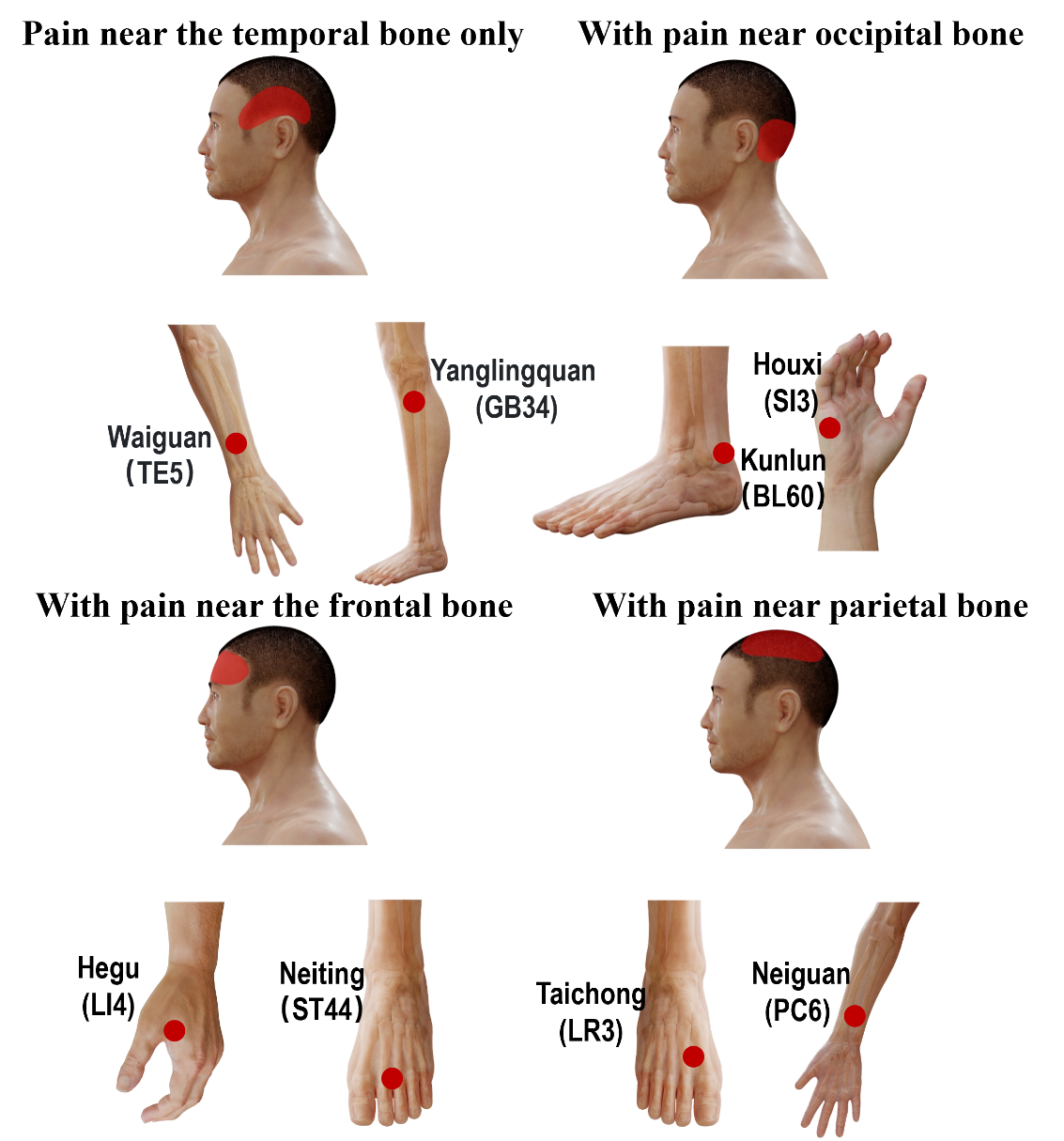


# Table S1. Outcome measurements of the per-protocol set population: headache diary-based outcome measurements during the entire study^a^

| **Variable** | **MPA (n=95)** | **NPA (n=85)** | **Difference, 95% CI** | ***P* Value** |
| --- | --- | --- | --- | --- |
| Primary outcome | | | | |
| Change from baseline in migraine attacks frequency at week 16^b^, mean (SD) | | | | |
| Week 16 | -2.6 (2.8) | -1.8 (2.8) | -0.7 (-1.6 to 0.09) | 0.080 |
| Secondary outcomes | | | | |
| Frequency of migraine attacks per 4 weeks^b^, mean (SD) | | | | |
| Baseline | 4.2 (2.6) | 4.3 (2.8) | -0.1 (-0.9 to 0.7) | 0.739 |
| Treatment, week 4 | 3.3 (2.0) | 4.0 (2.9) | -0.7 (-1.4 to 0.1) | 0.073 |
| After treatment | | | | |
| Week 8 | 2.1 (2.1) | 3.0 (2.5) | -0.9 (-1.5 to -0.2) | 0.014* |
| Week 12 | 1.8 (1.8) | 2.7 (2.3) | -0.9 (-1.5 to -0.3) | 0.002** |
| Week 16 | 1.6 (1.6) | 2.5 (2.1) | -0.9 (-1.4 to -0.3) | 0.002** |
| Proportion of responders per 4 weeks, No. (%) | | | | |
| Treatment, week 4 | 33 (34.7) | 17 (20.0) | 14.7 (1.9 to 27.5) | 0.018* |
| After treatment | | | | |
| Week 8 | 56 (58.9) | 35 (41.2) | 17.8 (3.4 to 32.2) | 0.025* |
| Week 12 | 63 (66.3) | 36 (42.4) | 23.9 (9.8 to 38.1) | 0.002** |
| Week 16 | 66 (69.5) | 40 (47.1) | 22.4 (8.3 to 36.5) | 0.003** |
| Days with migraine per 4 weeks^c^, mean (SD) | | | | |
| Baseline | 4.7 (3.2) | 4.7 (3.1) | 0.1 (-0.9 to 1.0) | 0.888 |
| Treatment, week 4 | 3.7 (2.7) | 4.5 (3.3) | -0.7 ( -1.6 to 0.2) | 0.105 |
| After treatment | | | | |
| Week 8 | 2.4 (2.4) | 3.3 (3.1) | -0.9 (-1.7 to -0.1) | 0.031* |
| Week 12 | 2.0 (2.1) | 3.1 (2.5) | -1.1 (-1.8 to -0.4) | 0.001** |
| Week 16 | 1.7 (1.9) | 2.8 (2.3) | -1.0 (-1.7 to -0.4) | 0.001** |
| VAS score per 4 weeks, mean (SD) | | | | |
| Baseline | 5.2 (1.3) | 5.2 (1.3) | 0.0 (-0.4 to 0.4) | 0.965 |
| Treatment, week 4 | 3.9 (1.4) | 4.4 (1.6) | -0.5 ( -1.0 to -0.1) | 0.017* |
| After treatment | | | | |
| Week 8 | 2.9 (2.0) | 4.0 (2.1) | -1.0 (-1.6 to -0.5) | <0.001*** |
| Week 12 | 2.7 (1.9) | 4.1 (2.0) | -1.3 ( -1.9 to -0.8) | <0.001*** |
| Week 16 | 2.6 (2.0) | 3.6 (2.1) | -0.9 (-1.5 to -0.3) | 0.003** |
| Use of acute pain medication per 4 weeks | | | | |
| Baseline | 45 (47.4) | 42 (49.4) | -2.0 (-16.7 to 12.6) | 0.785 |
| Treatment, week 4 | 26 (27.4) | 30 (35.3) | -7.9 (-21.5 to 5.6) | 0.253 |
| After treatment | | | | |
| Week 8 | 20 (21.1) | 25 (29.4) | -8.4 (-21.1 to 4.3) | 0.197 |
| Week 12 | 11 (11.6) | 23 (27.1) | -15.5 (-26.9 to -4.1) | 0.009** |
| Week 16 | 16 (16.8) | 20 (23.5) | -6.7 (-18.4 to 5.1) | 0.264 |

a. Data are given as mean (SD) except where noted; b. The frequency of migraine attack was defined as the number of episodes of migraine attack separated by pain-free intervals of at least 48h, as recorded in the headache diary; c. Number of days with migraine was defined as the duration of migraine attacks. **P*<0.05; ***P*<0.01; ****P*<0.001. MPA, manual acupuncture (manual penetrating acupuncture); NPA, placebo acupuncture (non-penetrating acupuncture); CI, confidence interval; VAS, visual analog scale; SD, standard deviation.

# Supplementary Table 2. Outcome measurements of the per-protocol set population: headache diary-HIT-6 score and MSQ score^a^

| **Variable** | | **MPA (n=95)** | **NPA (n=85)** | **Difference, 95% CI** | ***P* Value** |
| --- | --- | --- | --- | --- | --- |
| Secondary outcomes | | | | | |
| Change from baseline in HIT-6 Score, mean (SD) | | | | | |
| Baseline | 62.6 (7.0) | | 61.9 (7.3) | 0.8 (-1.3 to 2.9) | 0.470 |
| Treatment, week 4 | -7.0 (8.4) | | -4.8 (7.8) | -2.1 (-4.5 to 0.2) | 0.076 |
| After treatment | | | | | |
| Week 8 | -12.4 (11.1) | | -7.8 (10.0) | -4.4 (-7.5 to -1.4) | 0.005** |
| Week 12 | -13.7 (11.0) | | -7.8 (10.0) | -5.9 (-8.0 to -2.0) | <0.001*** |
| Week 16 | -14.0 (10.9) | | -9.8(10.1) | -4.2 (-7.3 to -1.1) | 0.007** |
| Change from baseline in MSQ score, role restrictive domain, mean (SD) | | | | | |
| Baseline | 60.1 (16.9) | | 61.2 (14.9) | -1.1 (-5.8 to 3.5) | 0.638 |
| Treatment, week 4 | 13.6 (19.4) | | 9.9 (17.6) | 3.8 (-1.6 to 9.2) | 0.167 |
| After treatment | | | | | |
| Week 8 | 20.3 (21.0) | | 12.5 (19.0) | 7.8 (2.0 to 13.6) | 0.009** |
| Week 12 | 21.5 (22.3) | | 13.1 (17.4) | 8.4 (2.6 to 14.2) | 0.005** |
| Week 16 | 23.0 (23.4) | | 15.7 (18.3) | 7.2 (1.1 to 13.4) | 0.020* |
| Change from baseline in MSQ score, role preventive domain, mean (SD) | | | | | |
| Baseline | 69.8 (19.3) | | 72.3 (19.1) | -2.5 (-8.1 to 3.1) | 0.383 |
| Treatment, week 4 | 12.2(20.9) | | 6.5 (17.3) | 5.6 (-0.04 to 11.2) | 0.048* |
| After treatment | | | | | |
| Week 8 | 16.0 (21.9) | | 8.5 (19.4) | 7.5 (1.4 to 13.5) | 0.015* |
| Week 12 | 16.8(23.1) | | 8.1 (18.5) | 8.7 (2.6 to 14.8) | 0.005** |
| Week 16 | 16.4 (23.6) | | 10.0 (19.8) | 6.4 (0.1 to 12.8) | 0.047* |
| Change from baseline in MSQ score, emotional functioning domain, mean (SD) | | | | | |
| Baseline | 77.4 (16.6) | | 77.0 (17.6) | 0.4 (-4.6 to 5.4) | 0.881 |
| Treatment, week 4 | 8.3 (20.6) | | 4.0 (16.8) | 4.3 (-1.2 to 9.7) | 0.125 |
| After treatment | | | | | |
| Week 8 | 10.1 (19.9) | | 5.8 (18.4) | 4.4 (-1.2 to 10.0) | 0.125 |
| Week 12 | 11.0 (22.8) | | 6.3 (18.8) | 4.7 (-1.4 to 10.7) | 0.132 |
| Week 16 | 10.6 (20.7) | | 9.1 (19.4) | 1.5 (-1.4 to 10.7) | 0.616 |

a. Data are given as mean (SD). **P*<0.05; ***P*<0.01; ****P*<0.001. MPA, manual acupuncture (manual penetrating acupuncture); NPA, placebo acupuncture (non-penetrating acupuncture); CI, confidence interval; HIT-6, Headache Impact Test-6; MSQ, Migraine-Specific Quality of Life Questionnaire; SD, standard deviation.

# Table S3. Baseline characteristics of patients with calcitonin gene-related peptide were measured

| **Characteristic** | **MPA (n=42)** | **NPA (n=21)** |
| --- | --- | --- |
| Age, mean (SD), year | 36.1 (10.3) | 35.1 (10.0) |
| Sex, No. (%) | | |
| Male | 10 (23.8) | 5 (23.8) |
| Female | 32 (76.2) | 16 (76.2) |
| BMI (Kg/m^2^), mean (SD) | 21.6 (3.1) | 21.5 (2.5) |
| Duration of illness, mean (SD), month | 97.7 (101.1) | 112.8 (115.9) |
| Degree of education, mean (SD), y | 15.3 (3.0) | 14.9 (3.6) |
| Family history, yes, No. (%) | 15 (35.7) | 5 (23.8) |
| Use of acute pain medication, No. (%) | 14 (33.3) | 7 (33.3) |

MPA, manual acupuncture (manual penetrating acupuncture); NPA, placebo acupuncture (non-penetrating acupuncture); BMI, body mass index; SD, standard deviation.

# Table S4. Change from baseline in calcitonin gene-related peptide

| **CGRP** | **MPA (n=42)** | **NPA (n=21)** | **Difference, 95% CI** | ***P* Value** |
| --- | --- | --- | --- | --- |
| Baseline, mean (SD) | 92.7 (25.2) | 95.4 (21.2) | -2.7 (-15.4 to 10.1) | 0.678 |
| Week 4, mean (SD) | -6.6 (15.1) | -0.2 (14.1) | -6.4 (-14.3 to 1.5) | 0.109 |
| Week 16, mean (SD) | -8.4 (26.2) | -1.8 (26.1) | -6.7 (-24.0 to 10.7) | 0.442 |

a. Data are given as mean (SD). CGRP, calcitonin gene-related peptide; MPA, manual acupuncture (manual penetrating acupuncture); NPA, placebo acupuncture (non-penetrating acupuncture); CI, confidence interval; SD, standard deviation.

**Table S5. Assessment of satisfaction in the intent-to-treat population**

| **Satisfaction for treatment** | **MPA (n=99)** | **NPA (n=99)** | ***P* Value** |
| --- | --- | --- | --- |
| Very dissatisfied, No. (%) | 0 (0.0%) | 1 (1.0%) | 0.001** |
| Less satisfied, No. (%) | 2 (2.0%) | 6 (6.1%) |  |
| General, No. (%) | 6 (6.1%) | 20 (20.2%) |  |
| Satisfactory, No. (%) | 31 (31.3%) | 33 (33.3%) |  |
| Very satisfied, No. (%) | 60 (60.6%) | 39 (39.4%) |  |

MPA, manual acupuncture (manual penetrating acupuncture); NPA, placebo acupuncture (non-penetrating acupuncture). ***P*<0.01.

# Table S6. Assessment of blinding of the intent-to-treat population

| **Blinding for treatment** | **MPA (n=99)** | **NPA (n=99)** | ***P* Value** |
| --- | --- | --- | --- |
| Manual acupuncture, No. (%) | 99 (100.0%) | 88 (88.9%) | 0.001** |
| Placebo acupuncture, No. (%) | 0 (0.0%) | 11 (11.1%) |  |

MPA, manual acupuncture (manual penetrating acupuncture); NPA, placebo acupuncture (non-penetrating acupuncture). ***P*<0.01.

# Table S7. Outcome measurements - participants with credible blinding: headache diary-based outcome measurements during the entire study ^a^

| **Variable** | **MPA (n=99)** | **NPA (n=88)** | **Difference, 95% CI** | ***P* Value** |
| --- | --- | --- | --- | --- |
| Primary outcome | | | | |
| Change from baseline in migraine attacks frequency at weeks 16^b^, mean (SD) | | | | |
| Weeks 16 | -2.6 (2.8) | -1.7 (2.7) | -0.8 (-1.6 to -0.04) | 0.040* |
| Secondary outcomes | | | | |
| Frequency of migraine attacks per 4 weeks^b^, mean (SD) | | | | |
| Baseline | 4.2 (2.5) | 4.2 (2.7) | 0.0 (-0.8 to 0.7) | 0.961 |
| Treatment, week 4 | 3.2 (2.1) | 3.9 (2.8) | -0.7 (-1.4 to 0.03) | 0.059 |
| After treatment | | | | |
| Week 8 | 2.1 (2.1) | 2.8 (2.5) | -0.7 (-1.4 to -0.05) | 0.036* |
| Week 12 | 1.7 (1.8) | 2.6 (2.2) | -0.9 (-1.5 to -0.3) | 0.004** |
| Week 16 | 1.6 (1.6) | 2.4 (2.1) | -0.9 (-1.4 to -0.3) | 0.002** |
| Proportion of responders per 4 weeks, No. (%) | | | | |
| Treatment, week 4 | 36 (36.4) | 16 (18.2) | 18.2 (5.7 to 30.6) | 0.007** |
| After treatment | | | | |
| Week 8 | 59 (59.6) | 39 (44.3) | 15.3 (1.1 to 29.5) | 0.038* |
| Week 12 | 67 (67.7) | 40(45.5) | 22.2 (8.3 to 36.1) | 0.003** |
| Week 16 | 68 (68.7) | 41 (46.6) | 22.1 (8.2 to 35.9) | 0.003** |
| Days with migraine per 4 weeks^c^, mean (SD) | | | | |
| Baseline | 4.7 (3.2) | 4.5 (2.9) | 0.2 (-0.6 to 1.1) | 0.611 |
| Treatment, week 4 | 3.7 (2.7) | 4.5 (3.3) | -0.8 (-1.7 to 0.1) | 0.073 |
| After treatment | | | | |
| Week 8 | 2.4 (2.4) | 3.1 (3.0) | -0.7 (-1.5 to 0.04) | 0.063 |
| Week 12 | 2.0 (2.1) | 3.0 (2.4) | -1.1 (-1.7 to -0.4) | 0.001** |
| Week 16 | 1.8 (2.0) | 2.7 (2.3) | -1.0 (-1.6 to -0.4) | 0.002** |
| VAS score per 4 weeks, mean (SD) | | | | |
| Baseline | 5.1 (1.3) | 5.3 (1.3) | -0.1 (-0.5 to 0.2) | 0.428 |
| Treatment, week 4 | 3.9 (1.5) | 4.4 (1.6) | -0.6 (-1.0 to -0.2) | 0.007** |
| After treatment | | | | |
| Week 8 | 2.9 (2.0) | 3.9 (2.1) | -1.0 (-1.6 to -0.4) | 0.001** |
| Week 12 | 2.7 (1.9) | 4.1 (2.0) | -1.3 (-1.9 to -0.8) | <.001*** |
| Week 16 | 2.7 (2.1) | 3.6 (2.2) | -0.9 (-1.6 to -0.3) | 0.002** |
| Use of acute pain medication per 4 Weeks, No. (%) | | | | |
| Baseline | 45 (45.5) | 44 (50.0) | -4.6 (-18.9 to 9.8) | 0.535 |
| Treatment, week 4 | 26 (26.3) | 30 (34.1) | -7.8 (-20.9 to 5.3) | 0.244 |
| After treatment | | | | |
| Week 8 | 20 (20.2) | 23 (26.1) | -6.0 (-18.1 to 6.2) | 0.337 |
| Week 12 | 11 (11.1) | 20 (22.7) | -11.6 (-22.3 to -0.9) | 0.034* |
| Week 16 | 16 (16.2) | 18 (20.5) | -4.3 (-15.4 to 6.8) | 0.448 |

a. Data are given as mean (SD) except where noted; b. The frequency of migraine attack was defined as the number of episodes of migraine attack separated by pain-free intervals of at least 48h, as recorded in the headache diary; c. Number of days with migraine was defined as the duration of migraine attacks. **P*<0.05; ***P*<0.01; ****P*<0.001. MPA, manual acupuncture (manual penetrating acupuncture); NPA, placebo acupuncture (non-penetrating acupuncture); CI, confidence interval; VAS, visual analog scale; SD, standard deviation.

# Table S8. Outcome measurements - participants with credible blinding: headache diary -HIT-6 score and MSQ score

| **Variable** | **MPA (n=99)** | **NPA (n=88)** | **Difference, 95% CI** | ***P* Value** |
| --- | --- | --- | --- | --- |
| Secondary outcomes | | | | |
| Change from baseline in HIT-6 Score, mean (SD) | | | | |
| Baseline | 62.3 (7.2) | 62.5 (7.1) | -0.2 (-2.3 to 1.9) | 0.848 |
| Treatment, week 4 | -6.7 (8.4) | -4.5 (7.7) | -2.2 (-4.5 to 0.1) | 0.060 |
| After treatment | | | | |
| Week 8 | -11.7 (11.3) | -8.1 (10.0) | -3.6 (-6.7 to -0.5) | 0.021* |
| Week 12 | -13.2 (11.3) | -8.2 (9.6) | -4.9 (-7.9 to -2.0) | 0.001** |
| Week 16 | -13.5 (11.3) | -10.0 (10.0) | -3.5 (-6.5 to -0.4) | 0.026* |
| Change from baseline in MSQ score, role restrictive domain, mean (SD) | | | | |
| Baseline | 60.7 (17.2) | 59.9 (15.9) | 0.8 (-4.0 to 5.5) | 0.745 |
| Treatment, week 4 | 13.4 (19.2) | 10.1 (18.3) | 3.3 (-2.0 to 8.7) | 0.225 |
| After treatment | | | | |
| Week 8 | 19.6 (21.1) | 14.6 (19.8) | 5.0 (-0.8 to 10.9) | 0.094 |
| Week 12 | 20.5 (22.8) | 14.6 (17.7) | 5.8 (0.01 to 11.6) | 0.049* |
| Week 16 | 22.2 (23.7) | 17.4 (19.9) | 4.8 (-1.5 to 11.0) | 0.132 |
| Change from baseline in MSQ score, role preventive domain, mean (SD) | | | | |
| Baseline | 70.4 (19.3) | 70.3 (20.1) | 0.01 (-5.6 to 5.7) | 0.997 |
| Treatment, week 4 | 11.7 (20.7) | 7.7 (19.1) | 4.0 (-1.7 to 9.7) | 0.171 |
| After treatment | | | | |
| Week 8 | 15.6 (21.9) | 10.5(20.3) | 5.0 (-1.0 to 11.1) | 0.102 |
| Week 12 | 16.7 (22.7) | 10.4 (20.3) | 6.3 (0.8 to 11.7) | 0.046* |
| Week 16 | 16.3 (23.3) | 11.9 (20.6) | 4.4 (0.1 to 12.4) | 0.172 |
| Change from baseline in MSQ score, emotional functioning domain, mean (SD) | | | | |
| Baseline | 77.8 (16.6) | 75.8 (17.3) | 1.9 (-2.9 to 6.8) | 0.434 |
| Treatment, week 4 | 8.3 (20.3) | 5.1 (17.4) | 3.2 (-2.2 to 8.6) | 0.245 |
| After treatment | | | | |
| Week 8 | 9.7 (19.6) | 6.4 (18.8) | 3.3 (-2.3 to 8.8) | 0.247 |
| Week 12 | 10.8 (22.5) | 7.5 (19.1) | 3.3 (-2.6 to 9.3) | 0.272 |
| Week 16 | 10.4 (20.5) | 9.9 (19.7) | 0.5 (-5.2 to 6.3) | 0.859 |

a. Data are given as mean (SD). **P*<0.05; ***P*<0.01; ****P*<0.001. MPA, manual acupuncture (manual penetrating acupuncture); NPA, placebo acupuncture (non-penetrating acupuncture); CI, confidence interval; HIT-6, Headache Impact Test-6; MSQ, Migraine-Specific Quality of Life Questionnaire; SD, standard deviation.
